# Rare Earth Hydroxide as a Precursor for Controlled Fabrication of Uniform β-NaYF_4_ Nanoparticles: A Novel, Low Cost, and Facile Method

**DOI:** 10.3390/molecules24020357

**Published:** 2019-01-19

**Authors:** Lili Xu, Man Wang, Qing Chen, Jiajia Yang, Wubin Zheng, Guanglei Lv, Zewei Quan, Chunxia Li

**Affiliations:** 1 Key Laboratory of the Ministry of Education for Advanced Catalysis Materials, Zhejiang Normal University, Jinhua 321004, China; 18329016701@163.com (L.X.); 15500100396@zjnu.edu.cn (M.W.); 1369976191@zjnu.edu.cn (Q.C.); 1547533672@zjnu.edu.cn (J.Y.); 13636130@zjnu.edu.cn (W.Z.); 2 Department of Chemistry, Southern University of Science and Technology, Shenzhen 518055, China

**Keywords:** β-NaYF_4_, rare earth upconversion nanoparticles, core–shell structure

## Abstract

In recent years, rare earth doped upconversion nanocrystals have been widely used in different fields owing to their unique merits. Although rare earth chlorides and trifluoroacetates are commonly used precursors for the synthesis of nanocrystals, they have certain disadvantages. For example, rare earth chlorides are expensive and rare earth trifluoroacetates produce toxic gases during the reaction. To overcome these drawbacks, we use the less expensive rare earth hydroxide as a precursor to synthesize β-NaYF_4_ nanoparticles with multiform shapes and sizes. Small-sized nanocrystals (15 nm) can be obtained by precisely controlling the synthesis conditions. Compared with the previous methods, the current method is more facile and has lower cost. In addition, the defects of the nanocrystal surface are reduced through constructing core–shell structures, resulting in enhanced upconversion luminescence intensity.

## 1. Introduction

In recent years, Lanthanide (Ln^3+^)-doped upconversion nanoparticles (UCNPs) that convert low energy photons into high energy photons through a two- or multi-photon absorption mechanism have extensively attracted researchers’ attention due to their potential applications in a variety of fields, such as bioimaging [1,2,3,4,5,6,7,8], biosensing [9,10], drug delivery [11,12], and cancer therapy [13,14,15,16]. Compared with traditional fluorescent probes, such as organic fluorescent dyes and semiconductor quantum dots, UCNPs possess some unique advantages, including weak background fluorescence, large anti-Stokes shift, high photochemical stability, narrow emission bandwidth, long luminescent lifetime, high penetration depth, and low toxicity, among others [17,18,19,20,21,22,23,24]. Among reported UCNPs, hexagonal phase (β-) sodium yttrium fluoride has been shown to be one of most efficient host materials owing to its low photon cutoff energy (~350 cm^−1^) and high chemical stability, which are able to effectively reduce non-radiative energy losses at the intermediate states of lanthanide ions [25]. So far, several methods have been reported to synthesize lanthanide-doped β-NaYF_4_ nanoparticles with controlled crystalline phase, shape, and size. Solvothermal method and thermal decomposition methods are two of the most frequently used techniques to synthesize monodisperse lanthanide-doped β-NaYF_4_ nanoparticles. For example, Haase reported the synthesis of β-NaYF_4_ by using expensive rare earth chlorides as precursors [26]. Capobianco and co-workers synthesized β-NaYF_4_ nanocrystals co-doped with Yb^3+^/Er^3+^ or Yb^3+^/Tm^3+^ via the thermal decomposition of rare earth trifluoroacetate precursors where octadecene (ODE) and oleic acid (OA) were chosen as a solvent and ligand, respectively [27]. However, there are disadvantages in this method, such as the high synthetic temperature, the complicated decomposition process, and the uncontrollable experimental conditions. More importantly, the heating of trifluoroacetate would produce toxic fluorinated and oxyfluorinated carbon gases.

In this paper, we developed a novel method by using cheap rare earth hydroxide as a precursor to synthesize monodisperse hexagonal NaYF_4_:Yb^3+^/Ln^3+^ core and NaYF_4_:Yb^3+^/Ln^3+^@NaGdF_4_ (Ln = Er, Tm, and Ho) core–shell nanoparticles with well-defined shapes. Compared with the previous methods, this method is low-cost and more facile. Moreover, during the reaction process no toxic gases are produced. In addition, the size of nanocrystals can be tuned by controlling the reaction conditions, such as the molar ratio of Na^+^/Ln^3+^/F^−^, the volume ratio of OA and ODE, and the amount of sodium oleate (NaOA). Under 980 nm laser excitation, these core–shell nanoparticles showed intense upconversion emissions relative to NaYF_4_:Yb/Ln (Ln = Er, Tm, and Ho) core nanoparticles.

## 2. Results and Discussion

**Figure 1:**
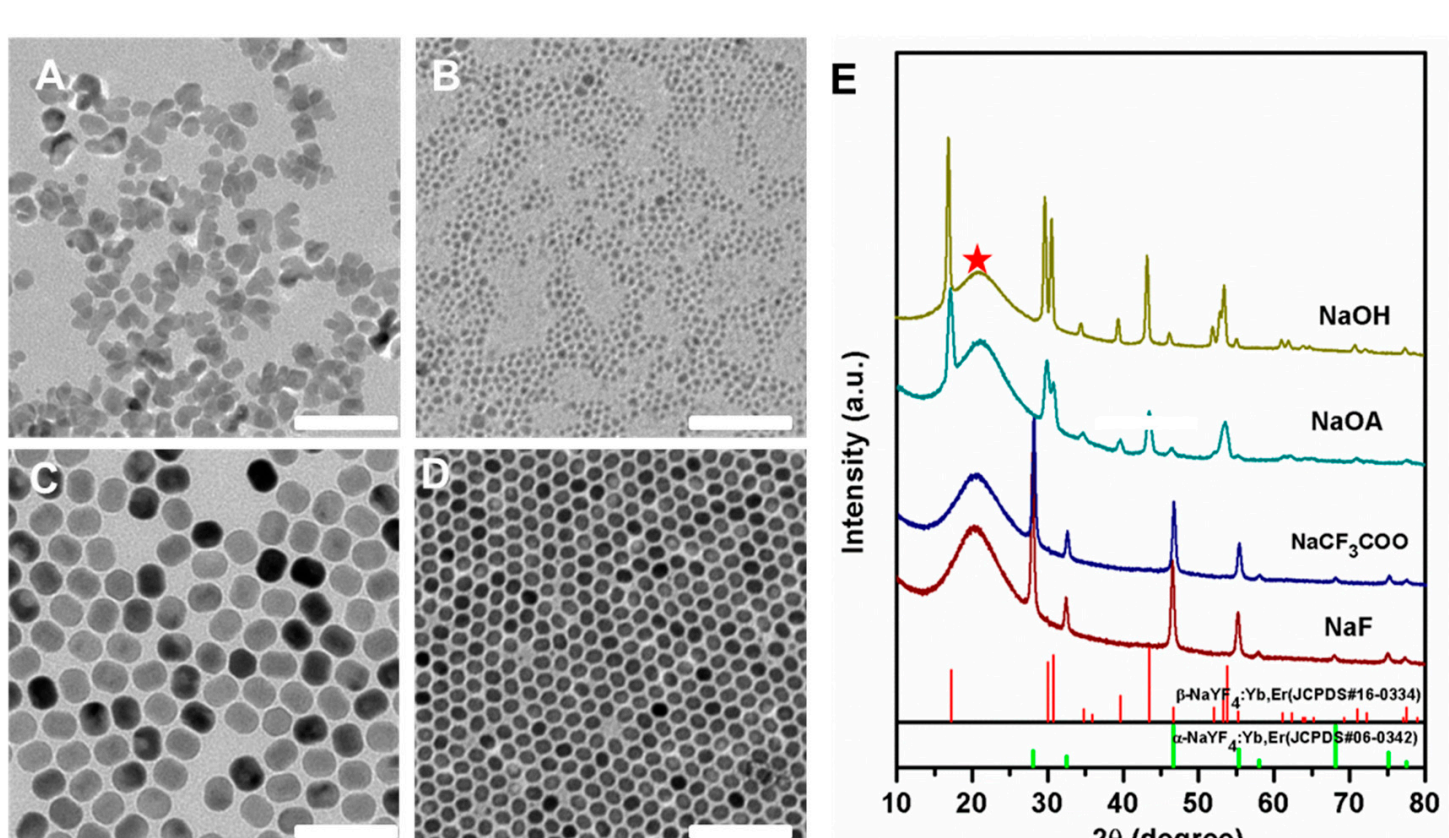
TEM images of NaYF_4_:Yb^3+^/Er^3+^ nanocrystals synthesized with NaOH (**A**), NaOA (**B**), CF_3_COONa (**C**), and NaF (**D**) as well as the corresponding XRD patterns (**E**). The red pentagram represents the peak of silica from the slide. The standard diffraction patterns of the α-NaYF_4_ (JCPDS 06-0342) and the β-NaYF_4_ (JCPDS 16-0334) are displayed at the bottom for reference. Scale bars, 100 nm.

It is well known that for the synthesis of nanomaterials, sodium sources and fluorine sources are two important factors in affecting the morphology and size of nanocrystals. In our experiments, we explored the effects of sodium hydroxide (NaOH), sodium oleate (NaOA), sodium trifluoroacetate (CF_3_COONa), and sodium fluoride (NaF) on the morphology and size of nanocrystals. It can be easily seen that the particle size was uneven when CF_3_COONa (Figure 1A) and NaF (Figure 1B) were used as sodium sources. Moreover, their powder X-ray diffraction (XRD) patterns were in line with the standard cubic one of JCPDS 06-0342 (Figure 1E). The broader width (red pentagram) of the XRD was ascribed to the peak of silica from the slide. However, NaYF_4_:Yb^3+^/Er^3+^ nanoparticles obtained with NaOH (Figure 1C) or NaOA (Figure 1D) as sodium sources had a uniform size and a crystal phase that matched well the standard JCPDS 16-0334 of β-NaYF_4_ (Figure 1E). In addition, the size of the upconverting nanoparticles synthesized with NaOA was smaller than that of NaOH. This means NaOA can effectively inhibit the growth of NaYF_4_ because of extra OA–ligands from NaOA. Taken together, the sodium source has a great influence on the size and crystal phase of the nanocrystals.

**Figure 2:**
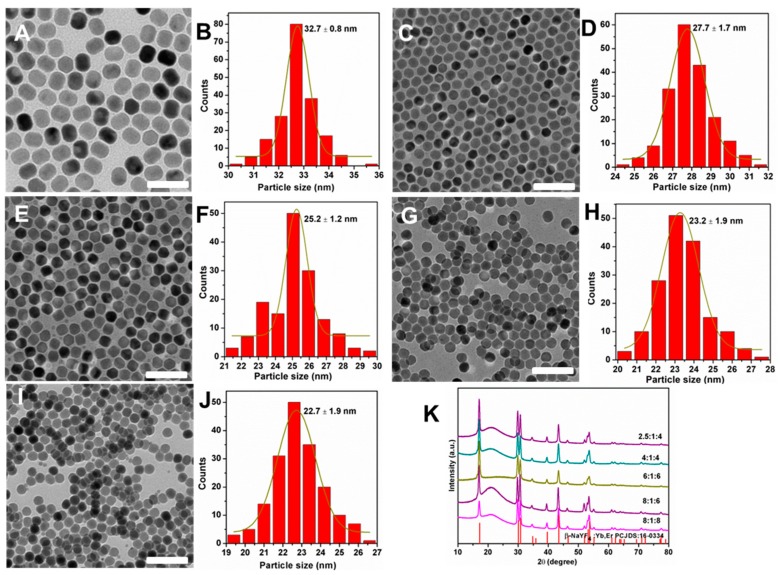
TEM images and size histograms of NaYF_4_:Yb^3+^/Er^3+^ nanocrystals synthesized with NaOH by using Na^+^/Ln^3+^/F^−^ with a molar ratio of 2.5:1:4 (**A**,**B**), 4:1:4 (**C**,**D**), 6:1:6 (**E**,**F**), 8:1:6 (**G**,**H**), and 8:1:8 (**I**,**J**), respectively, as well as the corresponding XRD patterns (**K**); the standard diffraction pattern of the β-NaYF_4_ (JCPDS 16-0334) is depicted at the bottom for reference. Scale bars, 100 nm.

Based on the above results, we firstly used NaOH as sodium sources to explore the effect of Na^+^/Ln^3+^/F^−^ on the size and morphology of nanocrystals. A series of NaYF_4_:Yb^3+^/Er^3+^ nanocrystals were synthesized with different ratios of Na^+^/Ln^3+^/F^−^ when the volume ratio of OA/ODE was fixed. Figure 2 presents the TEM images and size distributions of the NaYF_4_:Yb^3+^/Er^3+^ nanocrystals. It can be clearly seen that when the molar ratio Na^+^/Ln^3+^/F^−^ increased, the particle size of the products decreased accompanied by the shape evolution from rod to sphere. When the molar ratio of Na^+^/Ln^3+^/F^−^ was 2.5:1:4, the nanoparticles are regular rods with good monodispersity. Their lengths and widths are 32 nm and 27 nm (Figure 2A,B and Figure S1, Supporting Information), which are obtained by randomly measuring more than 150 particles. When the ratio of Na^+^/Ln^3+^/F^−^ was increased from 2.5:1:4 to 4:1:4, the size of the nanocrystals was reduced from 32.7 to 27.7 nm. It has been reported that the simultaneous addition of Na^+^ and F^−^ in the solution can produce small β-phase seeds, thereby the final growth of small-sized nanocrystals can be controlled easily [28]. Moreover, the size of the nanocrystals was decreased from 25 to 23 nm when the molar ratio of Na^+^/Ln^3+^/F^−^ was increased to be 6:1:6 (Figure 2G,H). When the molar ratio was further increased to 8:1:8 (Figure 2I,J), the size of the nanocrystals was reduced to 22 nm.

**Figure 3:**
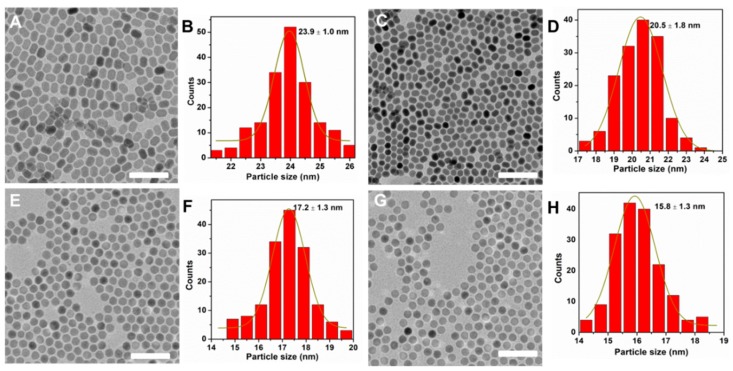
TEM images and size histograms of NaYF_4_:Yb^3+^/Er^3+^ nanocrystals synthesized with NaOA by using Na^+^/Ln^3+^/F^−^ withmolar ratios of 1.5:1:4 (**A**,**B**), 2.5:1:4 (**C**,**D**), 6:1:4 (**E**,**F**), and 8:1:4 (**G**,**H**), respectively. Scale bars, 100 nm.

To clarify the role of NaOA, we further studied the effect of its amount on the synthesis of NaYF_4_:Yb^3+^/Er^3+^ nanocrystals. Herein, we used NaOA substitute for NaOH to synthesize diverse nanoparticles with different molar ratios of Na^+^/Ln^3+^/F^−^. Figure 3 shows the TEM images of as-synthesized NaYF_4_:Yb^3+^/Er^3+^ nanocrystals, which exhibited the morphology and size evolution. As expected, the morphology of the nanocrystals changed from rods to spheres. Meanwhile, the size was reduced from about 24 to 16 nm. As the amount of NaOA increased from 1.5 to 2.5 mmol, the size of the nanocrystals decreased to 20 nm (Figure 3A–D). Then the size of NaYF_4_:Yb^3+^/Er^3+^ nanocrystals was further decreased to about 17 nm when the amount of NaOA increased to 6 mmol (Figure 3E,F) and 8 mmol (Figure 3G,H), respectively. It was possible that oxygen moiety in the OA–ligands had a much stronger binding affinity to Y^3+^ ions when there were adequate OA–ligands with the increased of NaOA.

**Figure 4:**
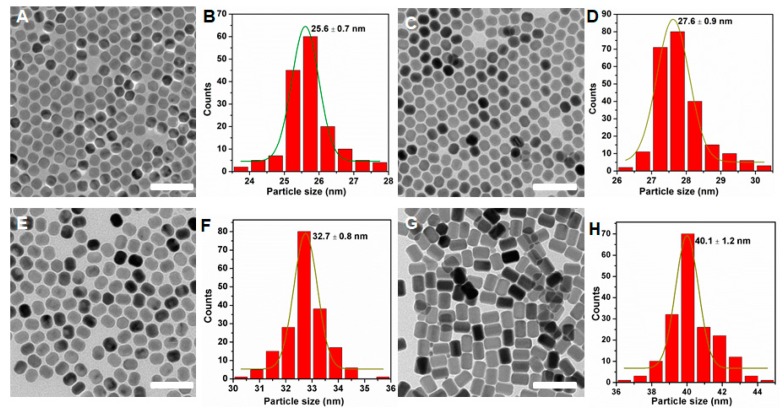
TEM images and size histograms of NaYF_4_:Yb^3+^/Er^3+^ nanocrystals synthesized at varied amounts of oleic acid (OA). The volume ratios of OA and octadecene (ODE) are 4:15 (**A**,**B**), 8:15 (**C**,**D**), 10:15 (**E**,**F**), and 15:15 (**G**,**H**), respectively. Scale bars, 100 nm.

The OA–ligands can effectively induce the orderly arrangement of Y^3+^ ions during the formation process of NaYF_4_ [29]. Additionally, when the amount of OA–ligands was adequate, they would cover the surface of the nanoparticles to inhibit the growth of nanocrystals, which is an effective method to obtain the nanocrystals with a smaller size. Figure S2 (Supporting Information) shows the XRD patterns of the as-prepared NaYF_4_:Yb^3+^/Er^3+^ core nanoparticles. All the XRD patterns of samples could be matched with the pure hexagonal-phases NaYF_4_ (JCPDS 16-0334), and no trace of other phases or impurities were observed, which clearly suggests the high crystallinities of these as-prepared nanoparticles. It has been reported that the presence of oleic acid in the solvent plays an important role in tuning the size and morphology of NaYF_4_:Yb^3+^/Er^3+^ nanocrystals [30]. Figure 4 shows the TEM images and size distributions of NaYF_4_:Yb^3+^/Er^3+^ nanoparticles prepared at different ratios of OA/ODE of 4/15 (Figure 4A,B), 8/15 (Figure 4C,D), 10/15 (Figure 4E,F), and 15/15 (Figure 4H,I). As can be seen, the resulting NaYF_4_:Yb^3+^/Er^3+^ nanoparticles with the ratio of OA/ODE (4/15) were hexagonal in shape with an average diameter of about 26 nm. With the increased volume ratio of OA/ODE, the particle size of NaYF_4_:Yb^3+^/Er^3+^ nanoparticles gradually increased, and the average diameter of nanoparticles was found to be approximately 40 nm at 8/15 of OA/ODE. The XRD of all as-prepared samples are shown in Figure S3 (Supporting Information). The same procedure was used to further synthesize NaYF_4_ nanoparticles doped with other lanthanide elements such as 40% Yb^3+^/0.5%Tm^3+^ (Figure S3A,B) and 18% Yb^3+^/2% Ho^3+^ (Figure S3C,D, Supporting Information), respectively. It was observed that the size and morphology of the nanoparticles closely resembled those of the Yb^3+^/Er^3+^ co-doped NaYF_4_ counterpart (Figure S1, Supporting Information). These results indicate that the change of dopant ions in low doping concentrations does not alter the particle growth process [31].

**Figure 5:**
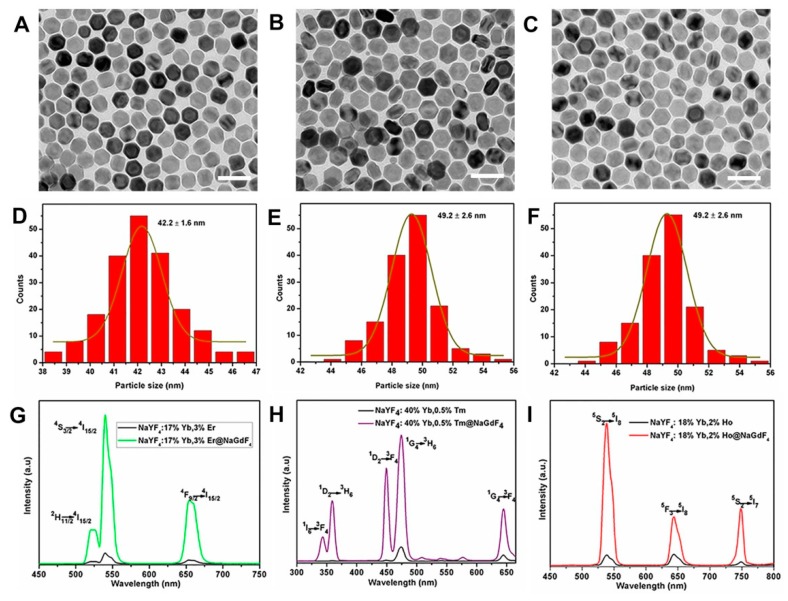
TEM images, size histograms, and the corresponding upconversion spectra of core–shell structured NaYF_4_:Yb^3+^/Er^3+^@NaGdF_4_ (**A**,**D**,**G**), NaYF_4_: Yb^3+^/Tm^3+^@NaGdF_4_ (**B**,**E**,**H**), and NaYF_4_:Yb^3+^/Ho^3+^@NaGdF_4_ (**C**,**F**,**I**), respectively. Scale bars, 100 nm.

More importantly, the emission intensity of Ln^3+^ doped UCNPs depends on dopant–host combination, particle size, shape, and phase [32,33,34,35,36,37,38]. The emission intensity of NaYF_4_ core was relatively weak due to the surface defects. However, in recent years, the construction of core–shell structure has been one of the effective ways to improve the efficiency of upconversion luminescence [26,39,40,41,42]. This is because the inert shell coating can protect the luminescent activators in the core nanoparticles from the surface quenching of excitation energy [42]. In this paper, we prepared NaYF_4_:Yb^3+^/Ln^3+^@NaGdF_4_ and studied their upconversion optical properties. Figure 5A–F displays the TEM images and the corresponding size distribution of NaYF_4_:Yb^3+^/Ln^3+^@NaGdF_4_ core–shell nanoparticles. The NaYF_4_:Yb^3+^/Er^3+^ core-only nanoparticles with an average diameter of about 27 nm are shown in Figure S1. After coating an inert shell layer of NaGdF_4_, the average diameter of NaYF_4_:Yb^3+^/Er^3+^@NaGdF_4_ core–shell was determined to be about 42 nm, which means a thick shell layer (thickness ~7.5 nm) was coated around the NaYF_4_:Yb^3+^/Er^3+^ core nanoparticles (Figure 5A,B). Similar TEM image and size distribution were obtained for NaYF_4_:Yb^3+^/Tm^3+^@NaGdF_4_ (Figure 5C,D) and NaYF_4_:Yb^3+^/Ho^3+^@NaGdF_4_ (Figure 5E,F) core–shell nanoparticles. The corresponding shell thickness was determined to be about 11 nm and 9 nm, respectively. Figure 5G depicts the upconversion emission spectra for the NaYF_4_:Yb^3+^/Er^3+^ core nanoparticles and the NaYF_4_:Yb^3+^/Er^3+^@NaGdF_4_ core–shell nanoparticles. As can be seen, there were two green emission bands centered at 520 nm and 540 nm, which were attributed to the electronic transition of ^2^H_11/2_ → ^4^I_15/2_, ^4^S_3/2_ → ^4^I_15/2_ of Er^3+^. In addition, there was a red emission at 654 nm, which corresponded to the ^4^F_9/2_ → ^4^I_15/2_ transition of Er^3+^. For the NaYF_4_:Yb^3+^/Er^3+^ core, after coating 7.5 nm NaGdF_4_ shell, the fluorescence intensity of NaYF_4_:Yb^3+^/Er^3+^@NaGdF_4_ core–shell nanoparticles was about 100 times higher than that of NaYF_4_:Yb^3+^/Er^3+^ core nanoparticles at 540 nm. In contrast, for the NaYF_4_:Yb^3+^/Tm^3+^ core nanoparticles, the blue emissions, the ultraviolet emission, and red emission corresponded to the (^1^I_6_ → ^3^F_4_, 342 nm, ^1^D_2_ → ^3^H_6_, 360 nm), (^1^D_2_ → ^3^F_4_, 450 nm, ^1^G_4_ → ^3^H_6_, 475 nm), (^1^G_4_ → ^3^F_4_, 647 nm) transitions of Tm^3+^ ions, respectively. Due to the NaGdF_4_ (~11 nm) shell coating, the emission intensity was remarkably enhanced to about 120 times at 475 nm (Figure 5G). Figure 5H shows the upconversion emission spectra of the NaYF_4_:Yb^3+^/Ho^3+^ core nanoparticles and the NaYF_4_:Yb^3+^/Ho^3+^@NaGdF_4_ core–shell nanoparticles. When excited at 980 nm, three upconversion fluorescence bands with maxima at green (540 nm), red (645 nm), and 750 nm regions could be observed from optimized NaYF_4_:Yb^3+^/Ho^3+^ nanophosphors, which corresponded to ^5^S_2_ → ^5^I_8_, ^5^F_3_ → ^5^I_8_, and ^5^S_2_ → ^5^I_7_ transitions of Ho^3+^ ions. Similarly, when the ~9 nm NaGdF_4_ shell was covered on the surface of the NaYF_4_:Yb^3+^/Ho^3+^ core, the luminescence intensity was also significantly improved. The emission intensity of NaYF_4_:Yb^3+^/Ho^3+^@NaGdF_4_ core–shell nanoparticles was determined to be about 80 times as strong as that of NaYF_4_:Yb^3+^/Ho^3+^ core nanoparticles.

## 3. Materials and Methods

### 3.1. Materials

Materials Y_2_O_3_ (99.99%), Yb_2_O_3_ (99.99%), Er_2_O_3_ (99.99%), Tm_2_O_3_ (99.99%), Ho_2_O_3_ (99.99%), Y_2_O_3_ (99.99%), GdCl_3_·6H_2_O (99.99%), NH_4_F (98%), NaF (98%), and CF_3_COONa (97%) were purchased from Aladdin (Shanghai, China). Oleic acid (OA, 90%), 1-octadecene (ODE, 90%), and sodium oleate (Na-OA, >97%) were purchased from Sigma-Aldrich (Darmstadt, Germany). Other chemical reagents, such as NaOH (90%), ethyl alcohol (99.7%), methanol (99.5%), and *n*-hexane (97%), were obtained from Shanghai Lingfeng Chemical Reagent Co., Ltd. All chemicals were used as received without further purification.

Rare earth chloride (LnCl_3_) stock solutions of 1 M (Ln = Y, Yb) and 0.1 M (Ln = Er, Tm, and Ho) were prepared by dissolving the corresponding metal oxides in hydrochloric acid at elevated temperature. The final solutions were adjusted to pH ~6.

### 3.2. Synthesis of β-NaYF_4_:Yb^3+^/Ln^3+^ (Ln = Er, Tm and Ho) Core Nanoparticles

First, Ln(OH)_3_ (Ln = Y^3+^, Yb^3+^, and Er^3+^/Tm^3+^/Ho^3+^) complexes were prepared by adding NaOH of 2 M to the rare earth chloride solution, and then the obtained product was washed twice. Rare earth hydroxide was added to a 100 mL flask containing 10 mL of OA and 15 mL of ODE. The mixture was heated at 140 °C for 1 h under stirring in order to form the lanthanide–oleate complexes. After cooling down to 40 °C naturally, 8 mL of methanol solution containing NH_4_F (4 mmol) and NaOH (2.5 mmol) was added. Subsequently, the resulting mixture solution was heated at 70 °C for 10 min to evaporate the methanol under magnetic stirring. After the temperature was raised up to 110 °C under vacuum for 30 min, the reaction mixture was heated to 300 °C under argon for 1 h and then cooled down to room temperature. The resulting nanoparticles were precipitated by adding excess ethanol, separated by centrifugation at 4000 rpm for 4 min, washed with a mixture of *n*-hexane and ethanol several times, and finally dispersed in *n*-hexane for further use.

### 3.3. Synthesis of β-NaYF_4_:Yb/Ln@NaGdF_4_ (Ln = Er, Tm, and Ho) Core–Shell Nanoparticles

In a typical procedure for the synthesis of NaYF_4_:Yb^3+^/Er^3+^ (17/3%)@NaGdF_4_ nanoparticles, the NaGdF_4_ inert shell precursor was prepared by mixing 0.5 mmol of GdCl_3_ with 6 mL of OA and 15 mL of ODE in a 100 mL flask followed by heating at 150 °C for 40 min. Then, the obtained NaGdF_4_ inert shell precursor was cooled down to room temperature. Subsequently, the NaYF_4_:Yb^3+^/Er^3+^ (17/3%) core-only nanoparticles dispersed in 3 mL of cyclohexane along with 8 mL of methanol solution of NH_4_F (2 mmol) and NaOH (1.25 mmol) was added. Subsequently, the resulting mixture solution was heated at 70 °C for 10 min under magnetic stirring to evaporate the methanol. After the resulting solution for 30 min at 110 °C under vacuum, the reaction mixture was heated to 310 °C under argon for 1.5 h and then cooled down to room temperature. Finally, the obtained core–shell nanoparticle products were precipitated by the addition of ethanol, collected by centrifugation at 4000 rpm for 4 min, washed with a mixture of *n*-hexane and ethanol several times, and finally dispersed in *n*-hexane.

### 3.4. Instrumentation

Transmission electron microscopy (TEM) images (Hitachi, Tokyo, Japan) were acquired on a HT7700 transmission electron microscopy at an acceleration voltage of 100 kV. X-ray diffraction (XRD) patterns were recorded on an X-ray diffraction (RigakuSmartLab, Tokyo, Japan) with Cu Kαradiation (λ = 0.15418 nm) at a voltage of 45 kV and a current of 20 mA. Upconversion luminescence spectra were detected by a spectrophotometer (FluoroMax-4, Shanghai, China) equipped with a 980 nm diode laser.

## 4. Conclusions

In summary, we developed a novel, facile, and low-cost method to synthesize Yb^3+^/Ln^3+^ (Ln = Er, Tm, and Ho) co-doped β-NaYF_4_ nanocrystals. The size and morphology of the products were manipulated through the precise tuning of the ratio of Na^+^/Ln^3+^/F^−^, the ratio of OA/ODE, and the quantity of the NaOA. As the ratio of Na^+^/Ln^3+^/F^−^ and the amount of NaOA increased, the size of the nanocrystals gradually decreased and the corresponding morphologies evolved from nanospheres to nanorods. Finally, this method can also apply to the fabrication of core–shell structured nanomaterials. The uniform NaYF_4_:Yb^3+^/Ln^3+^@NaGdF_4_ core–shell nanoparticles were prepared successfully, and their emission intensity was remarkably enhanced, which could provide prospect applications in the biomedical fields. Particularly, some photosensitizers or chemotherapy drugs could be conjugated with these UCNPs to achieve NIR-triggered drug delivery and controlled release to ameliorate the therapy efficiency of tumors.

## Supplementary Materials

The following are available online, Figure S1: TEM image (A) and the corresponding size histogram (B) of NaYF_4_:Yb^3+^/Er^3+^ nanocrystals synthesized with NaOH as sodium source, Figure S2: XRD patterns of the as-synthesized NaYF_4_:Yb^3+^/Er^3+^ nanocrystals with NaOA at varied molar ratios of Na^+^/Ln^3+^/F^−^. The standard diffraction patterns of the β-NaYF_4_ (JCPDS 16-0334) depicted at the bottom for reference, Figure S3: XRD patterns of the as-synthesized NaYF_4_:Yb^3+^/Er^3+^ nanocrystals at varied amounts of oleic acid. The volume ratios of oleic acid and octadecene are 15:15, 10:15, 8:15, and 4:15, respectively. The diffraction pattern at the bottom is the literature reference for hexagonal NaYF_4_ nanocrystal (JCPDS 16-0334), Figure S4: TEM images and size histograms of the NaYF_4_:Yb^3+^/Er^3+^ (A, B), and NaYF_4_: Yb^3+^/Ho^3+^ (C, D), respectively.
