# Supplementary material for: Rare Earth Hydroxide as a Precursor for Controlled Fabrication of Uniform β-NaYF4 Nanoparticles: A Novel, Low Cost, and Facile Method
Source: Molecules. 2019 Jan 19;24(2):357. doi: 10.3390/molecules24020357 (PMC6359501; doi:10.3390/molecules24020357)
Supplement: Supplementary file 1 [file molecules-24-00357-s001.pdf]

Rare Earth Hydroxide as Precursor for Controlled Fabrication of Uniform β-NaYF₄ Nanoparticles: A Novel, Low Cost and Facile Method

Lili Xu [1], Man Wang [1], Qing Chen [1], Jiajia Yang [1], Wubin Zheng [1], Guanglei Lv [1,*], Zewei Quan [2,*] and Chunxia Li [1,*]

[1] Key Laboratory of the Ministry of Education for Advanced Catalysis Materials, Zhejiang Normal University, Zhejiang 321004, China; 18329016701@163.com (L.X.); 15500100396@zjnu.edu.cn (M.W.); 1369976191@zjnu.edu.cn (Q.C.); 1547533672@zjnu.edu.cn (J.Y.); 13636130@zjnu.edu.cn (W.Z.)

[2] Department of Chemistry, Southern University of Science and Technology, Shenzhen, Guangdong 518055, China

* Correspondence: guanglei@zjnu.edu.cn (G.L.); quanzw@sustc.edu.cn (Z.Q.); cxli@zjnu.edu.cn (C.L.); Tel.: +86-0579-82282269 (G.L. and C. L.); +86-0755-88018399 (Z.Q.)

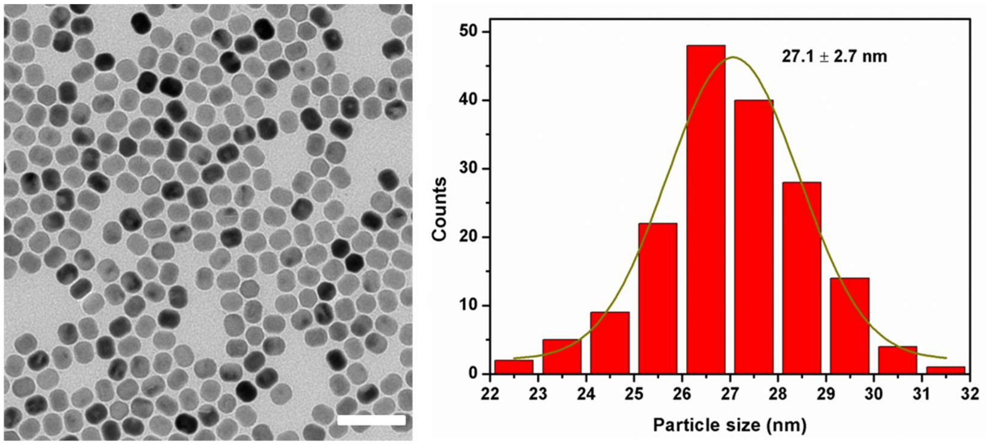

Figure S1 TEM image (A) and the corresponding width histogram (B) of $NaYF_4{:}Yb^{3+}/Er^{3+}$ nanocrystals synthesized with NaOH as sodium source. Scale bars, 100 nm.

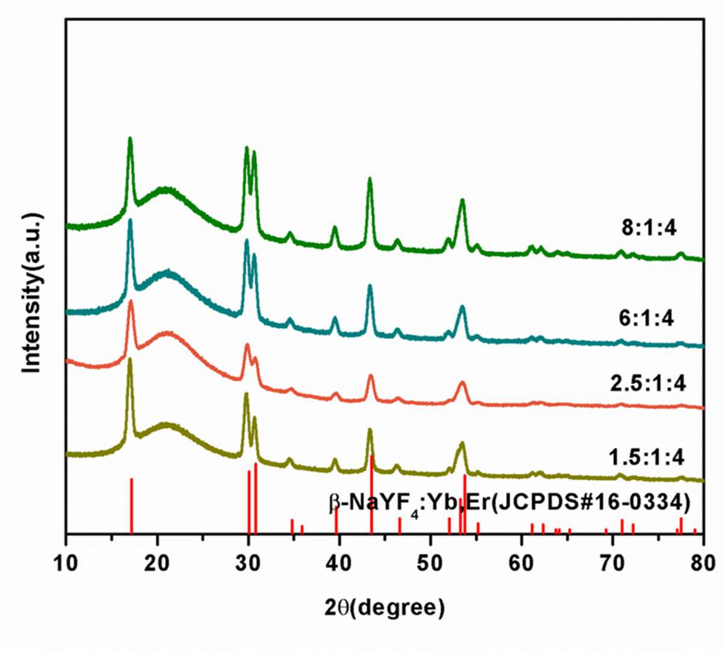

Figure S2 XRD patterns of the as-synthesized $NaYF_4:Yb^{3+}/Er^{3+}$ nanocrystals with NaOA at varied molar ratio of $Na^+/Ln^{3+}/F^-$. The standard diffraction patterns of the β-$NaYF_4$ (JCPDS16-0334) depicted at the bottom for reference.

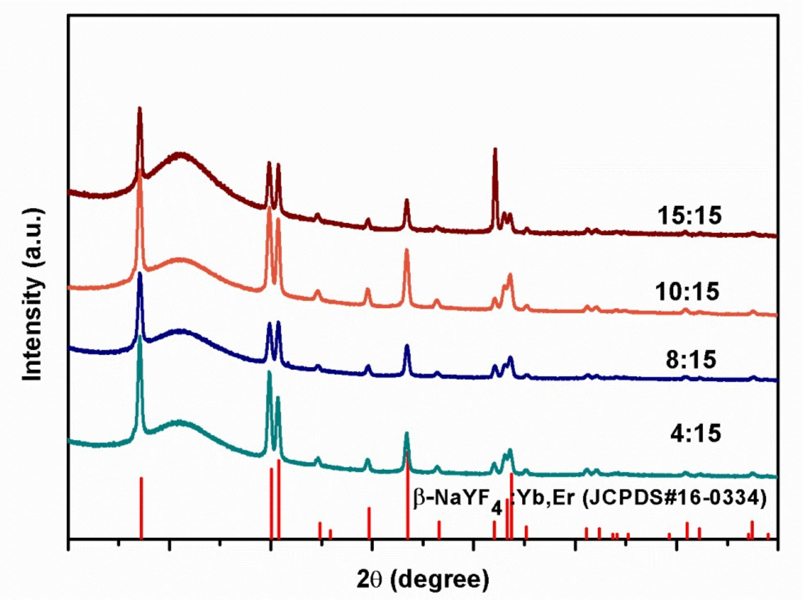

Figure S3 XRD patterns of the as-synthesized $NaYF_4:Yb^{3+}/Er^{3+}$ nanocrystals at varied amounts of oleic acid. The volume rations of oleic acid and octadecene are 15:15, 10:15, 8:15, and 4:15, respectively. The diffraction pattern at the bottom is the literature reference for hexagonal $NaYF_4$ nanocrystal (JCPDS16-0334).

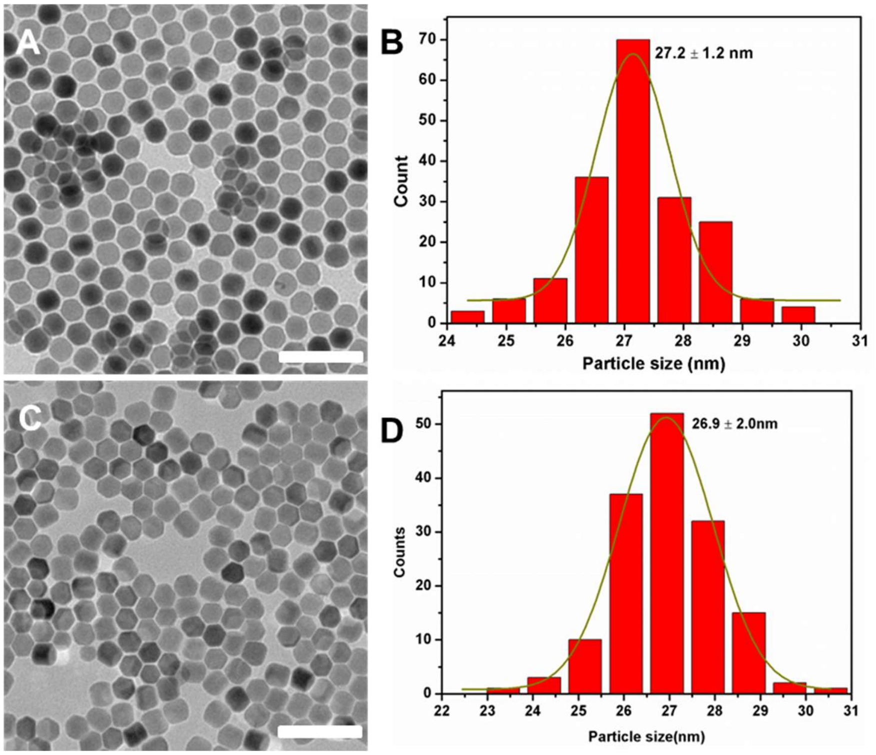

Figure S4 TEM images and size histograms of the $NaYF_4$:Yb^{3+}/Tm^{3+} (A, B) and $NaYF_4$: Yb^{3+}/Ho^{3+} (C, D), respectively. Scale bars, 100 nm.
